# Psychostimulants for Children: Are We Over or Under Dosing?

**Published:** 2023-04-22

**Authors:** Kenneth Blum, Catherine Dennen, Paul R. Carney, Elizabeth Gilley, Panayotis K. Thanos, Eric R. Braverman, David Baron, Colin Hanna, Edward J. Modestino, Mark S. Gold, Igor Elman, Rajendra D. Badgaiyan

**Affiliations:** 1 Division of Addiction Research & Education, Center for Sports, Exercise & Mental Health, Western University Health Sciences, Pomona, CA, USA; 2 The Kenneth Blum Institute of Neurogenetics & Behavior, LLC., Austin, TX, USA; 3 Department of Family Medicine, Jefferson Health Northeast, Philadelphia, PA, USA; 4 Division Pediatric Neurology, University of Missouri, School of Medicine, Columbia, MO, USA; 5 Behavioral Neuropharmacology and Neuroimaging Laboratory on Addictions, Clinical Research Institute on Addictions, Department of Pharmacology and Toxicology, Jacobs School of Medicine and Biosciences, State University of New York at Buffalo, Buffalo, NY, USA; Department of Psychology, State University of New York at Buffalo, Buffalo, NY, USA; 6 Department of Psychology, Curry College, Milton, MA, USA; 7 Department of Psychiatry, Washington University School of Medicine, St. Louis, MO, USA; 8 Cambridge Health Alliance, Harvard Medical School, Cambridge, MA, USA; 9 Department of Psychiatry, Mt. Sinai School of Medicine, New York, NY, USA

**Keywords:** ADHD, Genetics and epigenetics, Genome-wide association studies, Candidate genes, Methamphetamine, Dopamine and risk polymorphisms

## Abstract

An estimated 3% to 10% of school children meet the DSM-V criteria for ADHD (Attention-Deficit/Hyperactivity Disorder), however, to be over-diagnosed, the rate of children inappropriately diagnosed with ADHD (false positives) would have to be larger than the number of children with ADHD who are under-identified and not diagnosed (false negatives). Accordingly, a number of investigators take the position that under-treatment with psychostimulants, especially in children and adolescence, will result in continued ADHD symptomatology including future Substance Use Disorder (SUD). However, other researchers and clinicians believe otherwise and espouse laudable arguments for caution and prolonged methamphetamine treatment. While there is ongoing controversy of the role of genetics and epigenetics linked to ADHD, it seems clear that a number of dopaminergic genes and their risk polymorphisms act as DNA antecedents impacted by epigenetic induced methylation. Our hypothesis and literature review suggest that one possible solution is to embrace non addictive interventions to induce global dopamine homeostasis.

## Introduction

An estimated 3% to 10% of school children meet the DSM-V criteria for ADHD, however, to be over-diagnosed, the rate of children inappropriately diagnosed with ADHD (false positives) would have to be larger than the number of children with ADHD who are under-identified and not diagnosed (false negatives). Based on the review of research on factors that affect diagnostic accuracy and prevalence studies, Sciutto and Eisenberg concluded that claims that ADHD is systematically over-diagnosed cannot be justified [1]. Along similar lines, Froehlich et al. [2] provided some interesting prevalence data on ADHD in the United States (US). Of an estimated 2.4 million US children aged 8 to 15 years, 8.7% met the DSM-IV criteria for ADHD. Of those, 47.9% had been diagnosed previously, and 32.0% of those were consistently treated with ADHD medications during the preceding year. Boys were more likely than girls to be identified, and the wealthiest children from the highest quintile were significantly less likely than children from the lowest quintile to fulfill ADHD criteria. Poor children were less likely to receive consistent pharmacotherapy and less than half of the children who met the criteria reported having received either a diagnosis or treatment. More recent work from this group following a review of the literature reported that they could not find convincing evidence that any genetic variant or pharmacogenetic test has revealed clinical utility in pinpointing the optimal ADHD medication for a given individual patient, highlighting the need for further investigation [3].

## Methamphetamine Dosing and Diagnostic Criteria

Keeping these studies in mind, Boland et al. [4], in over 40 studies suggested that the majority suggest a robust protective effect of ADHD medication treatment on substance use disorders, accidents and injuries mood disorders, suicidality, criminality, traumatic brain injuries, motor vehicle crashes, and educational outcomes. Similarly, a meta-analysis demonstrated a protective effect of medication treatment on academic outcomes, accidents and injuries, and mood disorders [4]. Accordingly, their findings further indicate that ADHD medication treatments are linked with reduction in the risks for a wide range of ADHD-associated functional outcomes supporting efforts targeted at early diagnosis, possibly genetic testing [5] and treatment of people with ADHD.

The question of over prescription of stimulant medication to ADHD patients has been evaluated in a non-biased study by Jensen et al. [6-9]. They examined epidemiological survey data obtained from 1,285 children and their parents across four US communities. Across the pooled sample of children 5.1% met full DSM-III-R ADHD criteria. During the previous 12 months, 12.5% of those children who met the criteria had been treated with stimulants. Stimulants had also been prescribed for some children who, although they did not fully meet ADHD diagnostic criteria, were presented with high levels of ADHD symptoms, suggesting that this stimulant prescription had been appropriate. Accordingly, from this study the undertreatment with stimulant medication of certain patient populations needs to be addressed.

## Pharmacokinetics of Methamphetamine

AOne key area that has been analyzed by toxicologists involves the pharmacokinetics of methamphetamine (Ritalin^®^). It is well-known that Methylphenidate has 2 chiral centers, but the drug used in therapy consists of only the threo pair of enantiomers. d-threo-Methylphenidate is more potent than the l-enantiomer. Moreover, methylphenidate is prescribed as a racemic mixture that undergoes stereoselective clearance. Methylphenidate is a short-acting stimulant with a duration of action of 1 to 4 hours and a pharmacokinetic half-life of 2 to 3 hours. Maximum drug concentration after oral administration occurs at about 2 hours. Additionally, the drug is absorbed well from the gastrointestinal tract and easily passes to the brain. Importantly, Methylphenidate is efficacious for short- or long-term treatment for children with ADHD. Its mechanism of action is not completely but it is established that the drug affects the release and reuptake of dopamine in the striatum [6].

## Co-morbid SUD

Importantly, investigations have established that, of individuals with continuing ADHD symptoms, up to 50% have a SUD, ADHD as a consequence represents an independent risk factor for substance abuse [7]. For example, 40% of adults with ADHD are nicotine dependent compared to 26% in the adult general population [7]. It is known that nicotine increases focus and enhances decision making [8]. A variety of the symptoms of ADHD may be similarly mitigated by other classes of drugs and substances of abuse. Impulsive behavior and poor judgment in social settings also increase the risk to substance use of people with ADHD. The development of substance abuse in adolescents with ADHD seems to be accelerated by an earlier age of onset, faster progress from alcohol to another drug of abuse, longer duration and a shorter interval between the onset and drug dependence. According to Sullivan and Rudnik-Levin the dysregulated and disruptive behavior of people with ADHD puts them at greater risk for treatment failure and can interfere with treatment access and response [7]. Moreover, more recently, Bilgi et al. [9], using a number of diagnostic tests such as Wender Utah Rating Scale, Adult ADD/ADHD Diagnosis and Evaluation Inventory, and Fagerstrom Nicotine Dependence Test that were administered to the participants concluded that “Considering the argument of ADHD being an independent risk factor for nicotine dependence, we think the co-occurrence of the smoking addiction and ADHD symptoms in the context of dopamine dysregulation is important in the clinical setting”.

These studies raise serious questions about who should be prescribed stimulant medication, especially if there is a pre-addiction genetic risk [10] in ADHD candidates. Currently, there are two camps-overdosing methamphetamine [11] or underdosing [12] the drug for our youth. The prior identification of children at risk for Psychoactive SUD or Reward Deficiency Syndrome (RDS) by genotyping for “reward” gene(s) polymorphisms seems attainable with the advent of the Genetic Addiction Risk Severity (GARS^®^) test [13].

## ADHD Subtypes

It is known that in order to reduce spurious diagnosis and over prescription of stimulant medication for ADHD in young children (as early as preschool) clinical scientists have sought to subtype ADHD into viable classifications. But even this logical approach has met with poor diagnostic outcomes [14]. Using the Reimherr Adult Attention Deficit Disorder Scale it was found that robust impairment in three of the four emotional domains reflected a symptom severity level equivalent to that of the inattentive factor. Moreover, 59% met this threshold, defining them as ADHD emotion dysregulation presentation, as opposed to 41% with ADHD inattentive presentation. Furthermore, Reimherr et al. [14] also suggested that cluster analysis validated these groups by generating similar clusters with 85% agreement regarding membership. ADHD and emotion dysregulation presentation thereof subjects presented with more childhood ADHD symptoms, adult symptoms of oppositional defiant disorder, and evidence of personality disorder. In fact, earlier work by Lahey et al. [15] revealed that although the Combined subtype and Inattentive subtype could be stable enough in younger children, to dissect groups for research, they were not stable enough to use clinically to assess individual children. They [15] observed that over time children rarely remain in the Hyperactive subtype, however, most moved to the Combined subtype in later years although some would not remain in either subgroup. The researchers further suggested that using continuous ratings of hyperactivity-impulsivity symptoms should be embraced as an alternative as a diagnostic qualifier to classifying the nominal subtypes of ADHD in DSM-V. In addition, this group also reported that both the World Health Organization’s International Classification of Diseases Hyperkinetic Disorder (ICD-10 HKD) and the DSM-IV’s classification (while old) of ADHD exhibited predictive validity over six years, however, children with impairment that related to persistent ADHD symptoms seem to be under-identified by the ICD-10 HKD supportive of the underdiagnosed arena.

## Genetic Aspects

Undoubtably, ADHD is a clinically heterogeneous disorder of impulsivity, inattention, and hyperactivity with an early onset in children Based on a consensus of the literature, it is prudent to conclude that there is a real need to improve early diagnosis of ADHD toward being less subjective and more biologically objective [16]. Specifically, ADHD based on GWAS (Genome-wide association studies) [17] is considered a highly heritable childhood behavioral disorder affecting 5% of children and 2.5% of adults (overall range from 3 - 10%). Common genetic variants contribute to ADHD susceptibility. Demontis et al. [17] reported a genome-wide association meta-analysis of 20,183 individuals diagnosed with ADHD and 35,191 controls that identifies variants surpassing genome-wide significance in 12 independent loci. While scrutiny of the article does not adequately describe the 35,191 controls (especially for elimination of all RDS behaviors that could lead to spurious results), otherwise the elegant GWAS results, and meta-analysis indicate 12 loci to associate with ADHD. The concluded that there was strong concordance with GWAS of quantitative population measures of ADHD symptoms supports that clinical diagnosis of ADHD is an extreme expression of continuous heritable traits. It is well-known that ADHD has substantial shared heritability with other mental disorders, contributing to comorbidity and considered a subtype of RDS [18]. Moreover, others [19] report evidence for association of ADHD with allelic variants of the dopamine beta-hydroxylase and dopamine receptor D2 (DRD2) genes. In addition, more recent evidence using enrichment analysis, dopamine receptor D2, brain derived neurotrophic factor, *HTRF1A*, and dopamine receptor D4 were recognized as important hub genes among the reported ADHD genes [20].

Tools available today certainly have come a long way towards recognizing ADHD as a disease in the US and other countries, but non-subjective diagnosis awaits further research. In particular, because family, twin, adoption, segregation analysis, and molecular genetic studies have shown that ADHD has a substantial genetic component, it would be beneficial to provide the clinician and patient with the informative prediction of a potential predisposition to ADHD by developing a validated ADHD gene panel. In fact, Sullivan and Rudnik-Levin [7] reviewed genetic studies examining the role of the DA D2 (DRD2) gene (highly associated with drug seeking behavior) in the etiology of ADHD. According to data results from molecular genetic studies Faraone and Biederman [21], cautiously suggest that the susceptibility to ADHD may be due to the presence of three polymorphic genes; the D4 DA receptor gene, the DA transporter gene, and the D2 DA receptor gene However, other genes also have been associated with ADHD. Moreover, Faraone and Biederman [21] also suggest that in order to apply appropriate psychostimulant dosing the clinician should take into account not only genetic aspects but epigenetic as well including adversity, low socioeconomic status, marital distress, and last but not least complications during pregnancy and delivery epigenetic influence on candidate gene expression. In fact, a better understanding of the involvement of molecular neurogenetic opioid, mesolimbic dopamine, and psychostimulant connections in “wanting” supports clinical decisions regarding psychostimulant dosing. While both scientific and clinical professionals search for a Food and Drug Administration approved treatment for ADHD the induction of dopamine homeostasis, via activation of the brain reward circuitry, offers treatment for underlying neurotransmitter functional deficits, potential prophylaxis, and support for beneficial therapeutic efforts [see review 22].

## Conclusion

It is our expert opinion that it seems important that physicians, parents, and teachers become educated as to the risks of stimulant medication and be provided with alternative non-stimulant, but effective new medications or non-addicting nutrition-based therapies. An estimated 3% to 10% of school children meet the DSM-V criteria for ADHD, however, to be over-diagnosed, the rate of children inappropriately diagnosed with ADHD (false positives) would have to be larger than the number of children with ADHD who are under-identified and not diagnosed (false negatives). Accordingly, a number of investigators take the position that under-treatment with psychostimulants, especially in children and adolescence, will result in continued ADHD symptomatology including future SUD. However, other researchers and clinicians believe otherwise and espouse laudable arguments for caution and prolonged methamphetamine treatment. While there is ongoing controversy of the role of genetics and epigenetics linked to ADHD, it seems clear that a number of dopaminergic genes and their risk polymorphisms function as DNA antecedents impacted by epigenetic induced methylation. Our hypothesis and literature review suggest that one possible solution is to embrace non addictive interventions to induce global dopamine homeostasis possibly via electronic therapy (H-Wave, PRTMS), and pro-dopamine regulation like KB220 as epigenetic repair.
